# Supplementary material for: Electrophysiological Approaches to Understanding Brain–Muscle Interactions During Gait: A Systematic Review
Source: Bioengineering (Basel). 2025 Apr 29;12(5):471. doi: 10.3390/bioengineering12050471 (PMC12108685; doi:10.3390/bioengineering12050471)
Supplement: Supplementary file 1 [file bioengineering-12-00471-s001.zip › bioengineering-3517760-supplementary.pdf]

JBI Critical Appraisal Tool for Analytical Cross-Sectional Studies

1. Were the criteria for inclusion in the sample clearly defined?
2. Were the study subjects and the setting described in detail?
3. Was the exposure measured in a valid and reliable way?
    - *To evaluate this question, we focused on the description of EEG and EMG set-up and processing*
4. Were objective, standard criteria used for measurement of the condition?
5. Were confounding factors identified?
6. Were strategies to deal with confounding factors stated?
7. Were the outcomes measured in a valid and reliable way?
    - *The connectivity measure was taken as the outcome measure for the risk of bias assessment*
8. Was appropriate statistical analysis used?

Supplementary Table S1. Risk of bias assessment

	Overall risk	Q1	Q2	Q3	Q4	Q5	Q6	Q7	Q8
Petersen et al., 2012	L	N	Y	Y	Y	Y	Y	Y	Y
de Tommaso et al., 2015	H	N	N	Y	U	N	N	U	U
Winslow et al., 2016	M	NA	Y	Y	Y	U	U	Y	Y
Brantley et al., 2016	M	NA	Y	Y	Y	U	U	Y	Y
Storzer et al., 2016	M	N	Y	Y	Y	Y	Y	U	Y
Artoni et al., 2017	L	N	Y	Y	Y	Y	Y	Y	Y
Roeder et al., 2018	L	N	Y	Y	Y	Y	Y	Y	Y
Jensen et al., 2018	L	N	Y	Y	Y	Y	Y	Y	Y
Jensen et al., 2019a (main study)	L	N	Y	Y	Y	Y	Y	Y	Y
Jensen et al., 2019b (control experiment)	L	N	Y	Y	Y	Y	Y	Y	Y
Günther et al., 2019	M	Y	Y	Y	Y	Y	U	Y	Y
Li et al., 2019	L	Y	Y	Y	Y	Y	Y	Y	Y
Spedden et al., 2019	L	Y	Y	Y	Y	Y	Y	Y	Y
Hoxha et al., 2019	H	Y	Y	U	N	N	N	N	N
Chen et al., 2019	M	Y	Y	U	Y	Y	Y	Y	Y
Short et al., 2020	M	Y	Y	Y	Y	U	U	Y	Y

Roeder et al., 2020	L	Y	Y	Y	Y	Y	Y	Y	Y
Yokoyama et al., 2020	L	Y	Y	Y	Y	Y	Y	Y	Y
Gennaro & de Bruin, 2020a	L	Y	Y	Y	Y	Y	Y	Y	Y
Gennaro et al., 2020b	L	Y	Y	Y	Y	Y	Y	Y	Y
Chen et al., 2021	M	N	Y	Y	Y	U	U	Y	Y
Wei et al., 2021	M	N	Y	Y	Y	U	U	Y	Y
Manuel Mayor-Torres et al., 2022	H	N	Y	Y	Y	N	N	Y	Y
Caffi et al., 2022	L	Y	Y	Y	Y	Y	Y	Y	Y
Zhao et al., 2022	M	N	Y	Y	Y	U	U	Y	Y
Arunganesh et al., 2022	H	N	Y	U	Y	N	N	Y	U
Roeder et al., 2023	L	Y	Y	Y	Y	Y	Y	Y	Y

Y = Yes; N = No; U = Unclear; NA = Not applicable; L = Low; M = Moderate; H = High.
